# Knowledge, Practices and Attitudes towards Silver Diamine Fluoride Therapy among Dentists in Japan: A Mixed Methods Study

**DOI:** 10.3390/ijerph19148705

**Published:** 2022-07-17

**Authors:** Hollis Haotian Chai, Sakura Kiuchi, Ken Osaka, Jun Aida, Chun-Hung Chu, Shiqian (Sherry) Gao

**Affiliations:** 1 Department of Stomatology, School of Medicine, Xiamen University, Xiamen 361005, China; htchai89@connect.hku.hk; 2 Faculty of Dentistry, The University of Hong Kong, Hong Kong, China; chchu@hku.hk; 3 Graduate School of Dentistry, Tohoku University, Sendai 980-8577, Japan; sakura.kiuchi.e2@tohoku.ac.jp (S.K.); ken.osaka.e5@tohoku.ac.jp (K.O.); aida.ohp@tmd.ac.jp (J.A.); 4 Graduate School of Medical and Dental Sciences, Tokyo Medical and Dental University, Tokyo 113-8510, Japan

**Keywords:** silver diamine fluoride, caries, children, older population, mixed methods research

## Abstract

In 2021, the World Health Organization included silver diamine fluoride (SDF) as an essential medicine to manage caries in adults and children. SDF was developed in the 1960s, but its use for children became unpopular in Japan because of the decline and low prevalence of early childhood caries. This mixed methods study explored the knowledge, practices and attitudes towards SDF therapy among dentists promoting fluoride use in Japan. It also investigated senior dentists’ perceptions of SDF therapy in Japan. The quantitative study invited all 173 members of the largest organisation promoting fluoride use in Japan to complete a web-based questionnaire survey. Sixty (60/173; 35%) dentists promoting fluoride use in Japan completed the questionnaire survey. All dentists knew of SDF treatment, and 50 (50/60; 83%) used SDF for dental care. Fifty-nine dentists (59/60; 98%) agreed that SDF therapy was simple and quick. All 60 dentists agreed that SDF was effective to arrest caries; 51 dentists (51/60; 85%) agreed that SDF was effective to prevent caries. Most dentists (51/60; 85%) were concerned about SDF’s unaesthetic staining. Fifty-seven dentists (57/60; 95%) used SDF to arrest primary posterior teeth, and 52 dentists (52/60; 87%) used SDF to arrest root caries. However, 25 dentists (25/60; 42%) did not use SDF to prevent caries in permanent teeth. The qualitative study interviewed 12 senior dentists using snowball sampling and achieved data saturation. The dentists opined that SDF therapy was effective, simple, painless, non-invasive and inexpensive. SDF is seldom used in Japan at present because of the unaesthetic black staining and the low prevalence of early childhood caries; however, it can regain popularity by arresting root caries in the aging population.

## 1. Introduction

The Japanese word ‘Ohaguro’ means ‘black-stained teeth’. In ancient times, Ohaguro was the custom of painting teeth black in Japan, the south-eastern part of China, Vietnam, India, Indonesia, Solomon and the Mariana Islands [1]. The painting material mainly consisted of ferric acetate or ferrous sulphate, which, when combined with plant tannin, formed a black coating on the teeth. Culturally, people considered Ohaguro as a kind of beauty treatment. In addition, they believed that Ohaguro prevented dental caries [1]. A couple of centuries ago, people practiced Ohaguro for cultural and health reasons. The earliest record of Ohaguro dates to the late-third century in Japan [1]. People with a higher social class and married women in Japan widely adopted Ohaguro. The custom was maintained until the Westernisation of Asian countries in the 1800s.

**Figure 1:**
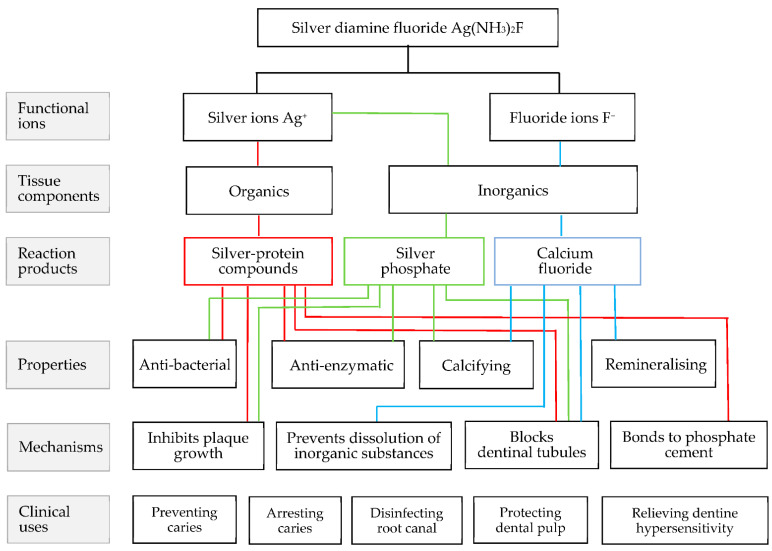
Functional ions, reaction products, properties, mechanisms and clinical uses of SDF per Yamaga.

Silver nitrate is a disinfectant. Dentists have been using silver nitrate to treat dental caries since the 1900s [2]. Fluoride effectively prevents dental caries and remineralises early enamel caries [3]. Using as inspiration the idea of Ohaguro and the cariostatic properties of silver nitrate and fluoride, professor Yamaga Reiichi from Osaka University used a painting material consisting of silver and fluoride for caries management. Since silver fluoride is unstable, ammonia was added to it to form silver diamine fluoride (SDF) [4]. SDF was prepared at 38% solution for dental treatment [5]. Since the 1960s, studies in Japan have reported that SDF effectively relieves hypersensitivity [6], prevents caries [7,8,9], arrests caries [10,11] and disinfects the root canal [10]. Figure 1 summarises the suggested functional ions and reaction products of SDF on tooth tissue, as well as the properties, mechanisms and clinical uses of SDF per professor Yamaga in the 1970s [7].

SDF was commercially available in Japan in the 1970s. Many dentists used SDF to treat dental caries in children in the 1970s and 1980s. According to the 1981 National Survey of Dental Diseases, dentists used SDF to treat 16% of the dental caries in 1 to 15-year-old children in Japan [12]. Apart from Japan, dentists in other countries such as Brazil and China also used SDF. A systematic review reported that SDF was effective in arresting caries [13]. In 2015, the United States Food and Drug Administration cleared SDF for dental use. In 2017, Health Canada approved SDF for dental care. In 2020, the British Society of Paediatric Dentistry published its support for the use of SDF to treat caries.

SDF therapy is an archetypal example of non-restorative caries treatment that steers sustainable caries management through controlling bacterial infection and remineralising teeth. With the paradigm shift of caries management from a surgical model to a medical model, an increasing number of clinicians advocate SDF therapy as a non-restorative approach for caries control. Since 2016, SDF has gained attention for managing dental caries worldwide. Regarding dental hypersensitivity management, the treatment ranges from non-invasive to in-office procedures. Studies have reported the effectiveness of calcium-fluoride-forming agent in comparison to fluoride varnish [14]. Evidence also shows that SDF is a safe treatment option for dental hypersensitivity [15]. In 2021, The World Health Organization included SDF as an essential medicine that is effective and safe to meet the most important needs in a health system for adults and children.

SDF has also attracted researchers’ attention since 2016. A recent bibliometric analysis found that the global research interest regarding SDF exponentially increased within the five years from 2016 to 2021 [16]. Furthermore, a steady increase in scientific research has been translated into clinical practice to improve the quality of dental care. Although SDF therapy has been gaining global popularity in recent years, it has become unpopular in Japan, and some young dentists have never used it. The objectives of this study were to explore the knowledge, attitudes and practices of SDF therapy for caries management among dentists who promote fluoride use in Japan, and to investigate the perception of SDF therapy among senior dentists working in universities and the government in Japan.

## 2. Materials and Methods

**Figure 2:**
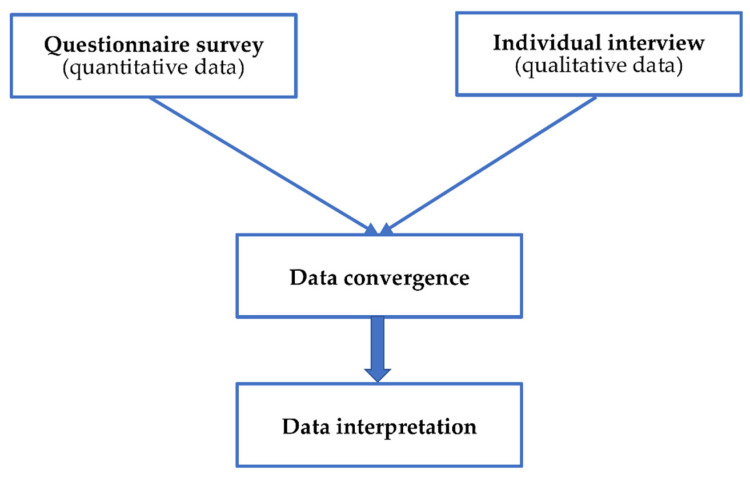
Concurrent triangular design of a mixed methods study.

The Ethics Committee of the Tohoku University Graduate School of Dentistry approved the study (No. 2018-3-8). This mixed methods study consisted of a quantitative study and a qualitative one. The quantitative study was an online questionnaire survey. The qualitative study was conducted using individual in-depth interviews. The study used the concurrent triangulation design, in which qualitative and quantitative data were collected concurrently (Figure 2).

### 2.1. Quantitative Study

The Japanese Association of Fluoride for Caries Prevention (www.nponitif.jp, accessed on 16 July 2022) is the largest association in Japan promoting fluoride use for preventing dental caries. The study invited all members of the association to complete a questionnaire survey. Table 1 shows the questionnaire’s content.

The questionnaire collected participants’ demographic information, including dental education backgrounds, dental practice and current position. It also collected the participants’ knowledge, practices and attitudes regarding SDF therapy:

1. Knowledge of SDF therapy: obtained by asking the participants when and where they learned about SDF and whether they included SDF in their teaching of dental students, if they have teaching activities;
2. Behaviour of practising SDF therapy: obtained by asking the participants about their SDF therapy practice;
3. Attitude towards SDF therapy: obtained by asking the participants their view regarding the effectiveness, advantages and disadvantages of SDF therapy.

The questionnaire in English was developed and discussed bya research group that was experienced in SDF therapy and dental public health. Two independent bilingual English and Japanese speakers translated the questionnaire from English to Japanese. Another two independent bilingual translators translated the Japanese draft back to English. The translators then compared the back-translated English version to the original English version to evaluate the semantic equivalence. They made further revisions according to the semantic equivalence’s results to develop the questionnaire in Japanese.

The questionnaire was pilot tested with 10 Japanese dentists. The translators finalised the questionnaire according to the 10 dentists’ comments. The questionnaire was then configured in a web-based version and presented bilingually in English and Japanese. Table 1 shows the content of the questionnaire for quantitative study.

Saforide (BEE BRAND MEDICO DENTAL, Japan) is made in Japan. It is basically the only commercially available SDF product in Japan. Dentists in Japan are more familiar with the term Saforide than they are with SDF. Thus, the survey used the term Saforide to achieve a better understanding among the participating dentists [17].

In June 2018, an invitation email with an introduction of the study and a web-based questionnaire link was sent to 173 dentists of the Japanese Association of Fluoride for Caries Prevention. In August 2018, a reminder email was sent to the members. The responses were collected on an online platform. Two researchers exported the collected data to an Excel file. They conducted data cleaning and performed a descriptive analysis.

### 2.2. Qualitative Study

The qualitative study invited renowned dentists for individual in-depth interviews to explore the perception of SDF therapy among senior dentists working in the university and government in Japan. The researchers identified a list of renowned dentists who were involved in the research and/or clinical use of SDF from the literature. Six of them agreed to an individual interview.

This study adopted snowball sampling to recruit subsequent dentists for interviews. The last question in each interview was, ‘Could you please advise if there are any dentists who might have insightful opinions regarding SDF use?’ This strategy helped us to identify additional potential participants who were knowledgeable about and had skills in SDF therapy. The sampling procedure stopped when the researchers found that the collected information repeated that from previous interviews.

Two researchers pilot tested a structured English interview guide with five dentists who were not involved in the main study. Two pairs of independent translators translated the guide in Japanese using the forward–backward translation method. They evaluated the questions’ wording and the content relevance according to the five dentists’ comments. 

Table 2 summarises the interview guide and presents the domains of and the key and follow-up questions for the interviews.

The final guide for the interview consisted of the four domains, as follows:

1. Background information of the interviewee, including the interviewee’s position and when and where their basic dental training took place;
2. Knowledge about SDF, including the interviewee’s learning and teaching experience;
3. Attitudes towards SDF use, including the interviewee’s views on the effectiveness, advantages and disadvantages of SDF therapy;
4. Behaviours of practicing SDF therapy, including the interviewee’s clinical practice of SDF application and its frequency of application.

**Figure 3:**
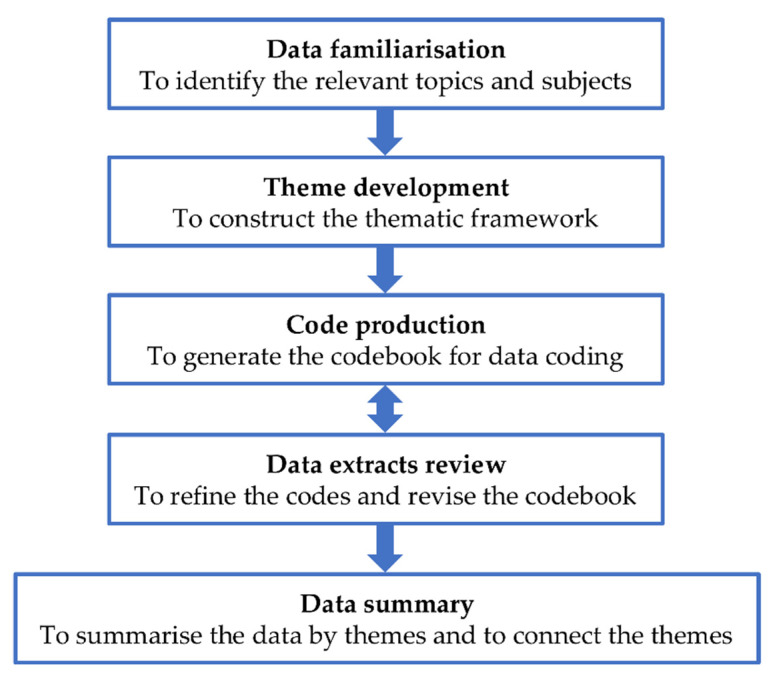
Data-managing process of the individual interviews.

The researchers took field notes and audio recorded the interviews. Throughout the study, the investigators and the researchers had regular meetings to evaluate the data that were collected and to discuss the progress. The interview continued until data saturation. A designated trained research assistant transcribed verbatim the audio records into a Word document. Two researchers performed a data analysis using the thematic approach. Figure 3 shows the five steps of the data-managing process of the individual interviews. The data-managing process of the individual interviews consisted of five steps:

1. Data familiarisation: Throughout the study, the researchers continued to read and re-read the interview transcripts. This process allowed them to become familiar with the data through identifying relevant topics and subjects;
2. Theme development: After identifying the relevant topics and subjects, the researchers refined and sorted them. This process allowed them to develop a set of themes and subthemes to construct the thematic framework;
3. Code production: Based on the developed thematic framework, the researchers independently and manually coded the transcripts. After coding every two transcripts, they discussed and evaluated them to reach an agreement on the produced codes. This process allowed them to generate a codebook for data coding;
4. Data extracts review: Using the codebook as a guide, the researchers regularly reviewed the data extracts to refine the codes . They repeated steps 3 and 4 to revise the codebook;
5. Data summary: Using the codebook, the researchers summarised the data according to the themes that were constructed. They also looked for the connections and associations between the themes.

## 3. Results

### 3.1. Quantitative Study

This study’s researchers sent an invitation email to all 173 members of NPO NichiF. Table 3 shows the participants’ demographic information.

Sixty members (60/173; 35%) accepted the invitation and completed the questionnaire. All respondents received their basic dental training in Japan; the majority (73%; 44/60) were trained before 1990. More than half of the dentists (32/60; 53%) held additional postgraduate dental degrees. The majority (48/60; 80%) first learned about SDF in their basic dental training. Thirty-two participants taught dental students; most of them (25/32; 78%) introduced SDF therapy to their students.

Almost all the dentists (59/60; 98%) agreed that SDF therapy was a simple and time-saving strategy for caries control. The major disadvantage was its black staining on the treated carious teeth or surfaces such as skin, clothes and operation stations.

Two-thirds of the dentists (40/60; 67%) agreed that the unpleasant taste was also a disadvantage of SDF therapy. However, 13 dentists (13/60; 22%) considered that the high concentration of fluoride and silver or the safety issues would be concerns of using SDF. Table 4 summarises the 60 dentists’ views on the advantages and disadvantages of SDF therapy.

All 60 dentists had used SDF before. Fifty of them (50/60; 83%) still used it and the remaining 10 no longer used SDF (10/60; 17%). All 60 dentists agreed to use SDF to arrest caries; 51 dentists agreed (nine dentists were not sure) to use SDF to prevent caries. However, only around one-fourth were frequent users.

The great majority of the participants would consider using SDF for arresting caries in primary posterior teeth (57/60; 95%); primary anterior teeth (53/60; 88%); and root caries (52/60; 87%). Most participants (45/60; 75%) would use SDF for preventing early childhood caries. These practice behaviours matched with their perceptions of the effectiveness of SDF therapy.

Nearly all dentists (54/60; 90%) considered SDF as very effective in arresting dental caries, while 36 (36/60; 60%) considered it as very effective in preventing dental caries. Table 5 shows the attitudes on their use and practice of SDF therapy in clinical care. No statistically significant (*p* > 0.05) result was found in the bivariate analysis of dentists’ attitudes toward the use and practice of SDF therapy (Table 5) per the demographic characteristics of participating dentists (Table 1).

### 3.2. Qualitative Study

Two researchers facilitated 12 individual interviews. The researchers achieved data saturation after interviewing the eleventh dentist for the in-depth interviews. Eleven of them were in-person (face to face) interviews and the remaining one was a telephone interview. The participants were senior dentists who graduated in or before the 1990s, and one of them graduated in the 1960s. The two researchers took field notes and audio recorded the interviews. The duration of the interviews ranged from 28 min to 129 min with a mean time of 72 min. Table 6 shows the demographic information of the 12 interviewees for the qualitative study.

#### 3.2.1. SDF Knowledge

All the interviewees reported that they had learned about SDF in their basic dental training. The interviewees who taught dental students introduced SDF therapy to arrest early childhood caries and root caries and to relieve hypersensitivity. Although all the universities at which the interviewees lectured covered SDF therapy, only one university used SDF therapy in its clinical training of dental students.

*‘In XXX University, a senior clinician looked after ten final-year (dental) students. I am sure all of them were given guidance and instruction of how to use SDF in the clinic.’*

—Interviewee No. 5

*‘Recently, we taught the prevention caries and the maintainers of restorations. At that time, we must give a lecture of restorations for the aged people; it is very difficult to treat root caries. So, I teach the students to treat root caries with Saforide.’*

—Interviewee No. 4

#### 3.2.2. SDF Clinical Practices

The interviewees used SDF to prevent caries, arrest caries, treat hypersensitivity and disinfect the root canal. They also used SDF as an indirect pulp-capping material for managing deep caries. They had contradicting views on SDF therapy for caries prevention. Some believed that SDF should not be used to prevent caries because fluoride varnish was effective for caries prevention. Some believed that SDF was effective and used it to remineralise early pits and fissure caries.

*‘It is important to use SDF to prevent early pits and fissure caries. SDF is effective to remineralise very early caries.’*

—Interviewee No. 9

*‘Saforide has two indicators, one is hypersensitivity, another is to stop the progression of caries.’*

—Interviewee No. 2

*‘First, to arrest the caries; second, to clarify the carious region; third, to prevent hypersensitivity, there is a possibility to extend the longevity of restorations (i.e., GIC after SDF application).’*

—Interviewee No. 4

The interviewees agreed that SDF was very effective in arresting dental caries, including early childhood caries and root caries. They also indicated that caries arrest was associated with good plaque control, proper tooth brushing and restricting sugar intake. These were important factors affecting the success of SDF therapy. Therefore, oral health education for children and their parents, midwives and public health nurses, etc. was important for achieving successful caries arrest. Softened dentine in the carious lesion should not be removed for SDF therapy. In addition, these patients should attend regular dental examinations to monitor their caries status.

*‘The soft surface of SDF-treated caries will become black and hard. However, if the surface is covered by plaque, it will become soft again.’*

—Interviewee No. 11

*‘But I must emphasise that not only SDF application but also (oral health) education to the children and (their) mothers (are important to arrest caries). (Oral health) education is very important. (Oral health) education includes sweet control and tooth brushing every day.’*

—Interviewee No. 6

The interviewees also shared their views on the indications of SDF therapy to arrest caries. They suggested that using SDF to arrest caries in primary anterior teeth, root caries, caries at the cervical area, caries that were difficult to access, caries on an area that was challenging for plaque control and caries in areas where aesthetics were not important.

*‘Yes, of course I used SDF, particularly for hidden tooth surfaces, areas which are very difficult to see, such as the distal surface of a second molar…. We cannot directly see the cavity (in the proximal tooth surface). Even in a very small cavity, we have to sacrifice the occlusal surface and restore the tooth as class II restoration.’*

—Interviewee No. 2

*‘Not only used for the older people, but also for the adults in some cases. Without considering the age, if the patients have exposed dentine, it is good to consider.’*

—Interviewee No. 5

Apart from young children, the interviewees suggested using SDF for older people, institutionalised people, people with dementia, people with xerostomia, people with difficulties travelling to the dental clinic, people who were handicapped, people who were hospitalised and people who were unable to brush their teeth.

*‘My schoolmate works in dental school. He serves older people in nursing homes. He treated (long-term hospitalised) patients in hospitals. He applies SDF to patients in hospitals, disabled people and institutionalised older people. Those patients need help. Their caries risk can develop rapidly. For these people, the saliva flow decreases because of polypharmacy and they cannot brush well.’*

—Interviewee No. 4

*‘The most important is patient acceptance (and) family acceptance. If the patient cannot decide, like with dementia, we will consult with the family. After getting informed consent, I applied.’*

—Interviewee No. 2

The interviewees also suggested that SDF therapy was beneficial for developing countries because of their high caries prevalence and limited dental resources.

*‘I think SDF is valuable for the (people in) developing countries. During the development, the amount of sugar introduced in the food is increased and hence the sugar consumption increases. In this situation, more caries will be developed.’*

—Interviewee No. 9

#### 3.2.3. Attitudes towards SDF Therapy

The interviewees mentioned the advantages and disadvantages of SDF therapy. They considered that SDF therapy was simple, quick, non-invasive, inexpensive and painless. They stated that it alleviated anxiety and fear towards dental treatment. Patients accept SDF well. The interviewees discussed the disadvantages of SDF therapy.

*‘Easy to apply. Treating time is short.’*

—Interviewee No. 7

*‘Anaesthesia is not needed. Be able to apply to very young children.’*

—Interviewee No. 8

*‘The disadvantages are the taste and influence on the gingiva. The metallic taste is very bad. It always stains caries black; sometimes it causes (gingival tissue to develop) white, corrosion-like irritation, which is similar to (those irritations caused by) a self-etching primer. After 24 h, it naturally disappears.’*

—Interviewee No. 2

Some dentists considered the black staining as beneficial because it showed the extent of the caries lesion. The black staining allows the patients to see the inconspicuous caries in their mouth, and thus is a way to raise their oral health awareness.

*‘For children, (it is) not painful, and we can use the black colour as a material for education. After 2–3 months later…if a child had plaque on the SDF applied tooth, I did the education again. It is very useful for dental education by using the black colour.’*

—Interviewee No. 6

#### 3.2.4. The Development of SDF Use in Dentistry

All the interviewees believed that SDF therapy had become uncommon in Japan. In the 1970s, the prevalence of early childhood caries was high and the dental workforce was inadequate in Japan. Therefore, government dentists used the simple and quick SDF therapy to manage the prevalent caries of children.

*‘After university graduation, I worked as the only public health dentist in a district social welfare department. I had to treat caries for more than 300,000 people living in that district. There were 5000 three-year-old children in the district. I used SDF to control caries for these children.’*

—Interviewee No. 9

*‘There was a time when Japanese children had a lot of caries (around 30 years ago). But now, the needs are becoming less and less. The country is developed very well, and the children’s oral health care is better; therefore, children don’t have many caries and have no need to use Saforide.’*

—Interviewee No. 3

In the 1980s, the number of dentists in Japan increased, whereas the child population decreased. At the same time, the caries prevalence in young children decreased. There were enough dentists to treat early childhood caries using conventional restorative treatment, and the government health insurance covered the treatment cost. As a result, using SDF for arresting early childhood caries became uncommon.

*‘In the 1980s, the number of Japanese dentists was increasing. At present, there are 60,000 dental clinics in Japan. Because of the increase of dentists and the decrease of coronal caries since the 1990s, as well as the decrease of the child population in Japan, SDF use diminished.’*

—Interviewee No. 4

The interviewees believed that dentists could use SDF therapy to manage older adults’ root caries. Some dentists believed that SDF had a positive future because it is a pragmatic treatment for frail and old people. The interviewees also reported that the Japanese Society of Conservative Dentistry recommended SDF therapy to control root caries.

*‘The official Society of Geriatric Dentistry has started to develop the guidelines for using Saforide. Soon it will be released. Market of SDF is going to expand.’*

—Interviewee No. 2

*‘Saforide is very useful to manage the caries in older people. When we restore the cavity of the root caries, I used SDF like a caries detector to manage the caries. I have a positive opinion of using SDF in the future.’*

—Interviewee No. 4

*‘Because of the decrease of dental caries in the children, now I think it is very important to educate the young dentists for using SDF not only for children but also for the aged person.’*

—Interviewee No. 6

## 4. Discussion

This study is the first to explore dentists’ perspectives of SDF therapy in Japan. Clinicians in Japan have been using SDF for more than 50 years, since the 1960s, and their experience is valuable for understanding SDF use for dental care. This research used a mixed methods study design with a concurrent triangulation design. The data collection was convergent parallel. A descriptive analysis and thematic analysis were used to analyse the quantitative data and qualitative data, respectively. The quantitative findings enhanced or built on the qualitative findings and vice versa [18]. Combining the quantitative data and qualitative data was beneficial because the study generated detailed, contextualized insights of the qualitative data and the generalizable, externally valid insights of the quantitative data. As a result, the study provided a comprehensive understanding of dentists’ attitudes and practices of SDF therapy in Japan. Although a mixed methods study has notable advantages over quantitative and qualitative studies, it is time-consuming and expensive. Since individual researchers often do not have the skill set that is required to perform all aspects of the study, a mixed methods study requires a team of researchers that are experienced in both quantitative and qualitative methods.

This study has its limitations. First, the majority of the participants for the questionnaire survey and all the participants for the individual interviews were experienced dentists who started their career in or before 1990. The responses from these majority senior dentists could overshadow the data that were collected. However, dentists who graduated after 2000 probably did not use or even know about SDF. Furthermore, many interviewees were academics or academic-related dentists. Greater diversification of the study group outside the academic community would be beneficial. However, senior dentists in private practice were probably retired and could not be located in this study. Second, this study invited dentists who supported using fluoride for caries management and who therefore might have held positive opinions towards SDF therapy. However, the interviewees pointed out that plenty of effective fluoride agents are readily available for caries prevention and that SDF has its limitations. Therefore, the researchers could not consider all of the invited participants as holding a positive opinion and as supporting the SDF use for caries management. Third, only one-third of the invited dentists participated in the questionnaire survey, although a reminder email was sent to boost the response rate. The view of this proportion of dentists might not fully represent the view of the invited dentists and could be a source of bias. However, there was no agreed-upon minimum acceptable response rate. A 35% response rate in this study was considered satisfactory, according to Lindemann [19], who judged 33% as the average survey response rate. In addition, the response rate of the web-based questionnaire survey is often not high because people receive a lot of junk emails [20]. For the in-depth interviews, the researchers achieved data saturation after interviewing the eleventh dentist. Therefore, this qualitative study stopped after interviewing the twelfth dentist.

SDF can be used to treat dental hypersensitivity and an infected root canal. Some interviewees also incorporated SDF therapy into their teaching as a desensitizing agent and caries arresting agent. Current evidence has revealed the effectiveness and safety of SDF therapy in treating dental hypersensitivity with reductions in sensitivity persisting seven days after application [21]. Although, research also found that calcium-fluoride-forming agents and fluoride varnish were effective in managing dental hypersensitivity [14]. However, no study has compared the efficacy of SDF to these two agents in treating dental hypersensitivity. Most dentists used SDF for caries control, even though the FDA approved SDF as a desensitizing agent in the US. The same circumstance was observed in Japan from this study, meaning that dentists in Japan used SDF principally for arresting dental caries. There were few research publications before 2000. This study showed that all the participating dentists agreed that SDF was effective in arresting dental caries based on their clinical observations and experience. Scientific evidence in the past that shows SDF’s effectiveness for caries arrest is insufficient. Recently, several systematic reviews concluded that SDF was effective in arresting early childhood caries and root caries [13,22,23]. These evidence-based findings were consistent with the participants’ perspectives in this study.

A systematic review reported that more studies are required to confirm the effectiveness of SDF for caries prevention [24]. The review’s findings also corroborate this study’s findings. Some dentists who were unsure used fluoride varnish instead of SDF for caries prevention. Plenty of clinical trial studies reported that a 5% sodium fluoride varnish was effective for caries prevention [25]. Although 38% SDF (44,800 ppm) has a higher fluoride concentration than 5% sodium fluoride (22,600 ppm), no study thus far has compared the caries preventive effect of the two fluoride agents.

In general, the dentists in this study understand well SDF therapy’s advantages. Almost all the dentists mentioned simplicity as a notable SDF therapy advantage. Since SDF use is simple and does not require complicated armamentarium, dentists can deliver SDF therapy in outreach settings, such as schools, nursing homes and general hospitals. Moreover, in arresting caries, SDF therapy is non-invasive and painless. It helps to reduce patients’ dental anxiety and fear of dental care. A clinical study found that only 4% of the young children with caries were uncooperative during treatment and could not receive SDF therapy [26].

Almost all the dentists mentioned the staining on other surfaces as a notable disadvantage of SDF therapy. If SDF is heedlessly handled and it spills on the skin, particularly on the face, it causes patient complaint and dissatisfaction. The staining can last for more than one week, even though it does not cause any pain. SDF also stains clothes, dental instruments, the dental chair and the working table in the dental surgery. The staining can be permanent and affect the clinic’s image, which may cause patients to consider that the clinic has a less than satisfactory quality of care.

Moreover, SDF causes black staining on the carious lesions, including early enamel lesions. The black staining, particularly on the anterior front teeth, is unaesthetic, causing patient dissatisfaction. Some clinicians consider patient-generated priorities as important for quality improvement in dental care. These priorities do not necessarily relate to the quality improvement initiatives. Hence, some dentists consider that SDF therapy with caries arrest is still a failure if the patient is dissatisfied with the aesthetic outcome. This is why most Japanese dentists do not use SDF to treat early childhood caries, although SDF has a history of success in the control of early childhood caries in Japan.

Although the black staining is an aesthetic concern, this study found some dentists who believed the staining was beneficial. The staining shows the margin of dental caries, which allows dentists to identify the extent of the carious lesion to facilitate caries removal in restorative treatment. In addition, some dentists believed the black staining allows the patients to easily identify the inconspicuous caries in their mouth. This helps the dentist show the patient the carious lesion that is found during examination. If the treatment is caries arrest via SDF, the patient can easily identify the site and pay more attention regarding plaque control to the arrested lesion. This is important for caries control because the arrested lesion can become active if dental plaque accumulates [27].

Early childhood caries was prevalent in Japan in the 1960s. In addition, there was an inadequate dental workforce to manage early childhood caries. With the introduction of SDF therapy in community dental care in the 1970s, the prevalence of early childhood caries significantly decreased to a low level [28]. One review found that early childhood caries was prevalent in most Southeast Asian countries [29]. The majority of early childhood caries that were found remained untreated, causing pain and infection. The review also found that early childhood caries was a major oral diseases burden caused. The conventional treatment of early childhood caries was often unavailable and unaffordable. SDF use in Japan can be an example for other countries to control prevalent early childhood caries with success.

Although SDF therapy for managing early childhood caries is uncommon in Japan, the interviewees reported that the Japanese Society of Conservative Dentistry recommended SDF therapy to control root caries. Similar to many developed, Japan’s aging population is rapidly growing. According to Japan’s government statistics in 2020, 36 million people, or 29% of the population, were 65 or over. This is almost the population of Poland, and it makes Japan the country with the oldest population by far. Root caries in older adults is a burden of dental disease. SDF therapy can be a pragmatic strategy to arrest prevalent root caries in the growing older population in Japan. The Japanese Society of Gerodontology recommended SDF therapy to treat root caries, particularly for patients with difficulties in accessing dental care, such as bedridden patients and patients with dementia.

## 5. Conclusions

Senior dentists and dentists promoting fluoride use in Japan understood SDF therapy’s advantages and disadvantages. They opined that SDF therapy was effective, simple, painless, non-invasive and inexpensive. SDF therapy is a non-restorative treatment that complements the conventional restorative management of dental caries. Apart from young children, SDF therapy can be used for older adults, institutionalised or hospitalized people and people with dementia or xerostomia. Moreover, people with special needs or who have difficulties in accessing dental care may also benefit from SDF application. SDF therapy is useful for acclimatisation or confidence building for patients with extreme phobias, as it is non-aerosol generating and arrests or delays caries progression. Hence, it can be operated in a pandemic outbreak when access to dental care is limited. SDF therapy is also promising in areas and circumstances with limited dental resources. SDF is seldom used in Japan at present because of the unaesthetic black staining and the low prevalence of early childhood caries. However, it can regain its popularity to arrest root caries in the aging population.

## Figures and Tables

**Table 1:** Content of the questionnaire.

*Demographic data*
• Name, main practice (Private, government/institution)
Dental practice (General practice, specialist practice) Dental education (Basic training, advanced training)
• Year and university of graduation of basic dental degree
*Domain 1. Knowledge*
• Have you ever heard of SDF (Saforide)? (Yes, No–end of survey)
• Did you first learn about SDF (Saforide) before you become a dentist? (Yes, No)
• Have you ever taught SDF (Saforide) to dental students? (Yes, No)
*Domain 2. Practice*
• Have you ever used SDF (Saforide) to treat your patient in the clinic? (Yes, No)
• Are you still using SDF (Saforide) to treat your patient in the clinic? (Yes, No)
• How many patients did you treat with SDF in last month? (0, 1–3, 4–9, 10 or above)
Will you use SDF to manage caries in the following situations? Prevent caries in primary teeth (Always, Sometimes, Never) Prevent caries in permanent teeth (Always, Sometimes, Never) Arrest primary anterior caries (Always, Sometimes, Never) Arrest primary posterior caries (Always, Sometimes, Never) Arrest permanent anterior caries (Always Sometimes, Never) Arrest permanent posterior caries (Always, Sometimes, Never) Arrest root caries (Always, Sometimes, Never)
• Will you deliver SDF therapy to the following populations?
Preschool children (Always, Sometimes, Never) Primary school students (Always, Sometimes, Never) Secondary school students (Always, Sometimes, Never) People with mental disorders (Always, Sometimes, Never) Adults aged 18–64 (Always, Sometimes, Never) Older adults aged 65 or above (Always, Sometimes, Never)
Will you deliver SDF therapy to the following people with special needs? People with mental disorders (Always, Sometimes, Never) People with physical disabilities (Always, Sometimes, Never)
*Domain 3. Attitude*
Which of the following would you consider as advantages of SDF (Saforide)? Please give your answer to each of the following potential advantages. Simple (Agree, Neutral, Disagree) Short application time (Agree, Neutral, Disagree) Non-invasive (Agree, Neutral, Disagree) Inexpensive (Agree, Neutral, Disagree) Painless (Agree, Neutral, Disagree)
Which of the following would you consider as disadvantages of SDF (Saforide)? Please give your answer to each of the following potential disadvantages. Unaesthetic (Agree, Neutral, Disagree) Unpleasant taste (Agree, Neutral, Disagree) Stains items (Agree, Neutral, Disagree) Toxic (Agree, Neutral, Disagree) Harmful due to high fluoride content (Agree, Neutral, Disagree) Harmful due to high silver content (Agree, Neutral, Disagree)
• How effective is SDF (Saforide) as a strategy for preventing dental caries? (Effective, Effective sometimes, Not effective, Not sure)

**Table 2:** The domains of and key and follow-up questions for the interviews.

Key Question(s)	Follow-Up Question(s)
*Background information*	
B.1 When did you obtain your basic dental training?	• How long is the basic dental training?
B.2 At which school did you study for basic dental training?	• What is your highest education level attained?
B.3 What is your current position?	• In which department do you work?
*Domain 1. Knowledge*	
1.1 When did you first learn about SDF?	• Why do you pay attention to SDF?
1.2 Where did you first learn about SDF?	In what kind of curriculum did you learn about SDF? Through what kind of media did you learn about SDF?
1.3 What messages about SDF did you deliver?	In what kind of curriculum have you taught SDF therapy? What basic knowledge of SDF have you delivered? What clinical knowledge about SDF have you delivered?
*Domain 2. Practice*	
2.1 How do you use SDF in clinical practice?	• With what kinds of patients would you use SDF?
	• How often did you apply SDF to your patients?
*Domain 3. Attitude*	
3.1 How effective do you think SDF is in caries management?	How effective SDF is in arresting childhood caries? How effective SDF is in arresting adult caries? How effective d SDF is in arresting root caries?
3.2 What are the advantages and disadvantages of SDF therapy?	What are the merits of using SDF? What are the indications of using SDF? What are the limitations of using SDF? What are the contra-indications of using SDF?
3.3 What were the challenges or barriers of using SDF?	What are the clinical-related barriers of using SDF? What are the barriers in other aspects of using SDF?

**Table 3:** Demographic information of the participating dentists (*n* = 60).

Items	Categories	No. of Dentists (%)
Year of obtaining the basic dental degree	1990 and later	16 (27%)
	Before 1990	44 (73%)
Location of the university for basic dental training	Eastern Japan	39 (65%)
	Western Japan	21 (35%)
Main employment	Private practice	46 (77%)
	Government or institution	14 (23%)
Dental practice	General practice	38 (63%)
	Specialist practice	22 (37%)
Higher dental training	Yes	32 (53%)
	No	28 (47%)
Learned about SDF therapy in basic dental training	Yes	48 (80%)
	No	12 (20%)
Participation in teaching dentistry in university	Yes	32 (53%)
	No	28 (47%)
Teaching SDF to dental students	Yes	25 (78%)
	No	7 (22%)

**Table 4:** Dentists’ views on the advantages and disadvantages of SDF therapy (*n* = 60).

Items	Categories	No. of Dentists (%)
*Advantages*		
Simple	Agree	59 (98%)
	Neutral	1 (2%)
	Disagree	0 (0%)
Short application time	Agree	59 (98%)
	Neutral	1 (2%)
	Disagree	0 (0%)
Non-invasive	Agree	55 (92%)
	Neutral	3 (5%)
	Disagree	2 (3%)
Inexpensive	Agree	48 (80%)
	Neutral	10 (17%)
	Disagree	2 (3%)
Painless	Agree	56 (93%)
	Neutral	3 (5%)
	Disagree	1 (2%)
*Disadvantages*		
Staining on teeth, unaesthetic	Agree	51 (85%)
	Neutral	4 (7%)
	Disagree	5 (8%)
Staining on items	Agree	48 (80%)
	Neutral	6 (10%)
	Disagree	6 (10%)
Unpleasant taste	Agree	40 (67%)
	Neutral	17 (28%)
	Disagree	3 (5%)
Toxic	Agree	21 (35%)
	Neutral	18 (30%)
	Disagree	21 (35%)
Harmful due to high silver content	Agree	15 (25%)
	Neutral	30 (50%)
	Disagree	15 (25%)
Harmful due to high fluoride content	Agree	13 (22%)
	Neutral	29 (48%)
	Disagree	18 (30%)

**Table 5:** Dentists’ practice of SDF therapy (*n* = 60).

Use of SDF Therapy	Frequency of Use	No. of Dentists (%)
To prevent caries in primary teeth	Always	17 (28%)
	Sometimes	28 (47%)
	Never	15 (25%)
To prevent caries in permanent teeth	Always	9 (15%)
	Sometimes	26 (43%)
	Never	25 (42%)
To arrest primary anterior teeth caries	Always	19 (31%)
	Sometimes	34 (57%)
	Never	7 (12%)
To arrest primary posterior caries	Always	28 (47%)
	Sometimes	29 (48%)
	Never	3 (5%)
To arrest permanent anterior caries	Always	7 (12%)
	Sometimes	17 (28%)
	Never	36 (60%)
To arrest permanent posterior caries	Always	16 (27%)
	Sometimes	27 (45%)
	Never	17 (28%)
To arrest root caries	Always	25 (42%)
	Sometimes	27 (45%)
	Never	8 (13%)

**Table 6:** Demographic information of the 12 interviewees for the qualitative study.

No. ^#^	Gender	Practice Profile	Year Qualified as a Dentist	Specialty
1	Male	Professor	1970s	Community Dentistry
2	Female	Professor	1970s	Conservative Dentistry
3	Male	Retired from university	1960s	Prosthetic Dentistry
4	Male	Associate professor	1980s	Restorative Dentistry
5	Female	Professor	1980s	Restorative Dentistry
6	Female	Retired from university	1960s	Paediatric Dentistry
7	Male	Professor	1990s	Paediatric Dentistry
8	Male	Associate professor	1980s	Preventive Dentistry
9	Male	Government dentist	1970s	Dental Public Health
10	Male	Government dentist	1980s	Dental Research
11	Male	Professor	1980s	Paediatric Dentistry
12	Male	Retired from university	1980s	Preventive Dentistry

^#^ No. is list according to the sequence of date of the interview.

## Data Availability

The data sets generated and/or analysed during the current study are available from the corresponding author upon reasonable request.
